# The Ubiquity of Cross-Domain Thinking in the Early Phase of the Creative Process

**DOI:** 10.3389/fpsyg.2019.01426

**Published:** 2019-06-19

**Authors:** Victoria S. Scotney, Sarah Weissmeyer, Nicole Carbert, Liane Gabora

**Affiliations:** ^1^Department of Psychology, University of British Columbia, Kelowna, BC, Canada; ^2^School of Population and Public Health, University of British Columbia, Vancouver, BC, Canada

**Keywords:** creativity, cross-domain, domain-general, domain-specific, influence, inspiration

## Abstract

To what extent are creative processes in one domain (e.g., technology) affected by information from other domains (e.g., music)? While some studies of professional creators suggest that creative abilities are domain-specific, other studies suggest that creative avocations stimulate creativity. The latter is consistent with the predictions of the honing theory of creativity, according to which the iterative process culminating in a creative work is made possible by the self-organizing nature of a conceptual network, or worldview, and its innate holistic tendency to minimize inconsistency. As such, the creative process is not restricted to the creative domain; influences from domains other than that of the final product are predicted to impact the creative process and its outcome. To assess the prevalence of cross-domain influences on creativity we conducted two studies: one with creative experts, and one with undergraduate students from diverse academic backgrounds. Participants listed both their creative outputs, and the influences (sources of inspiration) associated with each of these outputs. In both studies, cross-domain influences on creativity were found to be widespread, and indeed more frequent than within-domain sources of inspiration. Thus, examination of the inputs to, rather than the outputs of, creative tasks supported the prediction of honing theory that cross-domain influences are a ubiquitous component of the creative process.

## Introduction

Creative thought is central to human life. It shapes everyday activities such as putting together an outfit or holding a conversation. It fuels cultural evolution, giving us technology, music, media, and art. It fosters a sense of personal and cultural identity. Yet although it is perhaps our most defining human trait, it is one of the most elusive aspects of human cognition.

Creativity is thought to involve the restructuring of information in a creative domain, sometimes referred to as the *problem domain* (Runco, 2014), or simply, the domain. *Domain-specific theories* of creativity emphasize the non-transferability of expertise from one creative domain to another (Baer, 2015). These theories appear to be supported by findings that creative individuals are rarely creative in more than a few domains (i.e., someone known for their creativity in physics is rarely also known for their creativity as a dancer; Kaufman and Baer, 2004a; Baer, 2012), and by low correlations between an individuals’ creative products in different domains (Baer, 1991). Domain-specific theories are consistent with evidence that revolutionary creativity–sometimes referred to as ‘Big C’ creativity (Kaufman and Beghetto, 2009)–requires specific knowledge and skills in a domain, which requires extensive practise and learning, a phenomenon referred to as the ‘10-year rule’ on the basis of evidence that 10 years’ experience in a domain is necessary for creative success (Ericsson et al., 1993). Furthermore, latent class analysis has demonstrated categorically distinct groups of creative achievement, furthering the argument for domain-specific creativity, especially in regard to creative products (Silvia et al., 2009).

*Domain-general theories* emphasize the generalizability of creative thinking across different domains (Hong and Milgram, 2010). The domain general view is supported by personality studies, which suggest that there is something to the notion of a creative personality type (Eysenck, 1993; Martindale and Daily, 1996; Feist, 1998; Batey and Furnham, 2006), and by evidence that when people express themselves in different creative domains these outputs bear a recognizable style or ‘voice’ (Gabora et al., 2012). These findings suggest that the creative mind seeks to explore and express its distinctive structure and dynamics using whatever means available.

In another study, when painters were instructed to paint what a particular piece of music would ‘look like’ if it were a painting, naïve participants were able to correctly identify at significantly above chance which piece of music inspired which painting (Ranjan, 2014). Although the medium of expression was different, something of its essence remained sufficiently intact for people to detect a resemblance between the new creative output and its inspirational source. This suggests that, at their core, creative ideas are less domain-dependent than is widely assumed. The study supported our intuitive conviction that even when the creative *output* lies squarely in one domain, the *creative process giving rise to it* may be rooted in different domains.

Currently, many scholars espouse a less dichotomous view of creativity that incorporates both domain-specific and domain-general elements (Kaufman and Baer, 2004b; Plucker and Beghetto, 2004; Gabora, 2017). Possible mechanisms underlying cross-domain creativity have been suggested (Runco, 2009; Simonton, 2009; Sternberg, 2009), and tested in empirical studies (Boccia et al., 2015; An and Runco, 2016; Barbot et al., 2016; Palmiero et al., 2016, 2019). However, we believe that the domain-generality of creativity is still under-appreciated due to emphasis on the final *product* or output of the creative process, and a relative paucity of research on the inspiration phase of the creative process. Even if creative individuals tend to express themselves in one domain, this does not necessarily mean that prior phases of their creative process are domain-specific. An exception to the paucity of research on creative inspiration is Root-Bernstein et al. (1995) and Root-Bernstein (1996, 2001, 2003) work on how cross-domain influences such as artistic avocations can stimulate creativity in accomplished scientists. Our research differs from Root-Bernstein’s ground-breaking work in that it aims to assess how widespread such influences are, and therefore it is not limited to individuals with established careers in one or more creative domains.

This paper describes two studies designed to test the hypothesis that cross-domain influences play a normal and natural role in creativity and that cross-domain thinking constitutes a ubiquitous part of the creative process. The studies investigate possible sources of inspirations that lead to a specific creative output in both creative experts and non-experts.

### Honing Theory

#### Central Aim and Core Concepts

These studies are part of a larger research program developed to test, the *honing theory of creativity* (HT). While the central aim of most theories of creativity is to account for the existence of creative products, arose to account for the cumulative, open-ended nature of cultural evolution. HT grew out of the view that humans possess two levels of complex, adaptive, self-organizing, evolving structure: an organismic level, and a psychological level (Freeman, 1991; Varela et al., 1991; Barton, 1994; Pribram, 1994; Combs, 1996; Gabora, 1998, 2017). We refer to this psychological level as a *worldview:* an individual’s unique dynamic web of understanding that provides a way of both *seeing* the world and *being in* the world (i.e., a mind as it is experienced from the inside). In short, HT posits that the worldview is the hub of a second evolutionary process—cultural evolution—that rides piggyback on the first—biological evolution—and that creative thinking fuels this second evolutionary process (Gabora, 1998, 2004, 2008, 2013, 2017).

Honing an idea entails reiteratively looking at it from the different angles proffered by one’s particular worldview, ‘putting ones’ own spin on it by making sense of it in one’s own terms, followed by expressing it outwardly (Gabora, 2017). Honing may involve the restructuring of representations by re-encoding the problem such that new elements are perceived to be relevant, or relaxing goal constraints (Weisberg, 1995). It may also involve self-organized criticality, wherein small perturbations can have large effects (Gabora, 1998). As the creator’s understanding of the task shifts, the creative idea may find a form that fits better with the worldview as a whole, such that the worldview achieves a more coherent state, as formalized by the notion of *conceptual closure* (Gabora, 2000; Gabora and Steel, 2017). Creative acts and products render such cognitive transformation culturally transmissible. Thus, it is suggested that what evolves through culture is not creative contributions but worldviews, and cultural contributions give hints about the worldviews that generate them.

#### Predictions of HT Concerning Cross-Domain Influences

HT posits that creative output reflects the idiosyncratic, transformative process by which a worldview restructures itself in response to perturbations such as the detection of threats, inconsistencies, ambiguity, or potentiality. Such perturbations cause *arousal-provoking uncertainty*, which Hirsh et al. (2012) refer to as *psychological entropy*. It has been proposed that psychological entropy can not only be experienced negatively as anxiety, but also positively as a force that energizes creativity, by setting self-organized iterative honing into motion (Gabora, 2017). HT posits that since the contents of the mind exist in a state of potentiality until evoked from memory, and the form they take when they actualize in conscious awareness is subject to modification due to context, none are *a priori* excluded from the creative task. Thus, mental contents can fluidly cross domain boundaries, and it is possible for the domain-specific aspects of an idea to be stripped away such that it is amenable to re-expression in another form.

Because a worldview can continuously renew its overall structure, there are no limits on the possible influences or ‘conceptual parents’ of a creative work such as a song or journal article. For example, consider the situation in which a book inspires a movie, which inspires an invention. To see the thread of continuity across this ‘line of descent’ it is necessary to consider how their creators navigated through webs of beliefs, attitudes, procedural and declarative knowledge, and habitual patterns of thought and action that emerged through the interaction between personality and experience. In short, HT predicts that cross-domain influences play a role in the creative processes that fuel the self-organized transformation of worldviews, and that this in turn is the driving force of cultural evolution.

### Previous Research on Creative Influences

There have been efforts to corroborate anecdotal reports of creative influences (see Feinstein, 2006) with machine learning techniques designed to resolve lines of influence (Saleh et al., 2014). However, these techniques are not yet able to discern cross-domain influences, wherein a creator in one domain (e.g., art) is influenced by another domain (e.g., music). The study of cross-domain influences on creativity has been somewhat limited and has yielded conflicting results (Chan and Schunn, 2015). Some researchers have found that problem solvers from outside the given domain often produce the most creative solutions (Wiley, 1998; Jeppesen and Lakhani, 2010; Franke et al., 2014). Other evidence suggests that directly relevant information about a problem domain can increase the usefulness of a creative solution, but indirectly relevant information may enhance novelty (Montag-Smit and Maertz, 2017). Furthermore, exposure to artwork in an unfamiliar style has led to increased creativity in later artwork for novice artists (Okada and Ishibashi, 2017). Chan et al. (2015) examined the conceptual distance of inspirational sources on the quality of design ideas, and found that conceptually closer sources (which were defined as sharing a ‘topic’ of closely associated words) were associated with higher quality solutions. However, by searching the ‘conceptual genealogies’ of concepts through analysis of cited inspirations from earlier stages in the development of the design (‘indirect’ sources), creative benefit came from indirect sources with higher conceptual distance (Chan and Schunn, 2015). Since the designers were not asked to provide all elements that inspired their work, the scope of study was limited to the kinds of influences that one might logically expect to have a direct bearing on the result. Thus, for example, they listed things like previously generated solutions or design ideas, but not things like a particular piece of music, or ‘a conversation with a friend.’

### The Present Studies

This paper describes two studies of the prevalence of cross-domain influence in real-world creative endeavors.^1^ The first study involves individuals who have achieved some degree of expertise and/or recognition for their creative work. The second was carried out with non-experts.

## Study 1

The goal of Study 1 was to provide a preliminary assessment of the extent to which creative outputs are inspired by within-domain influences (i.e., either directly related to the creator’s domain of creative expression, such as a painter’s painting inspired by another painting, or indirectly related to the creator’s domain of creative expression, such as a painter’s painting inspired by a photograph) versus cross-domain influences (i.e., unrelated to the creator’s domain of creative expression, such as a painter’s painting inspired by a piece of music). We hypothesized that the majority of participants’ creative outputs will be inspired by cross-domain influences rather than within-domain influences.

### Methods

#### Participants

An internet search was conducted to locate individuals who were highly creative in any domain. They were recruited by email and invited to participate in the study on a voluntary basis. The method and recruitment email were both approved by the UBC Research Ethics Board. A total of 151 individuals participated.

#### Procedure

Participants were provided a link to an online questionnaire hosted by SurveyMonkey. The questionnaire asked their gender, age, and occupation, as well as the following open-ended questions:

(1) What is the general category for the creative work for which you are most known (e.g., art, music, drama, science)?
(2) What is the subcategory for the creative work for which you are most known (e.g., painting, piano composition, biochemistry)?
(3) Please describe your creative outputs.
(4) Please describe as best you can your creative process.
(5) Describe all elements that have inspired your work (natural or artificial, or it may be a particular event or situation, or something not in the concrete environment, that is, something abstract that you have been thinking about), and with each item, if possible, put as much identifying information as you can about the item it inspired (e.g., my Sunlight Sonata in B Flat composed in 2012 was inspired by going skiing in the alps with my sister who had just recovered from pneumonia). Do this for as many of your creative works as you can, itemizing them as (a), (b), (c), and so forth. Provide as much detail as possible.

Question four was not used in this analysis, as we chose to focus only on the inspirational elements provided. The influences provided in response to question five were divided into four categories: *cross-domain* (C), *within-domain narrow* (WN), *within-domain broad* (WB), and *uncertain* (U). A response was classified as WN if the domain of inspiration and the domain of the creative output fell within the same subcategory (e.g., a painting inspired by another painting). An example of a WB influence is a painting inspired by a photograph. A photograph and a painting belong to the same domain of visual art, but not to the same sub-domain. Cross-domain influences were those for which the domain of the influence was different from the domain of creative expression. For example, a poem (domain: writing) that inspired a piano piece (domain: music) was rated as a cross-domain influence. An answer was categorized as U when insufficient information was provided to categorize the influence as WN, WB, or C. Examples of each category of influence in a few different domains of creative output are provided in Table 1. Participants did not provide examples for all domain-category combinations.

**TABLE 1:** Examples of categories of influence for different types of creative outputs (Study 1).

	**Categories of influence**			
**Creative output**	**WN**	**WB**	**C**	**U**
Painting	Other paintings	Spirograph	Global warming	Knowledge
Writing	Other writings	Fairytales	Bathroom keys	Scripture
Music	Melodies	–	Dream	–
Photography	Editorial shoots	Art exhibitions	Meditation	–

*WN, within-domain narrow; WB, within-domain broad; C, cross-domain; U, uncertain. A dash indicates no example was present for that category.*

The rationale for using the self-report method was that only the participants themselves would be able to know what inspired their creative outputs. Outside assessments were not used in this study (or the next) since we were not interested in the quality of their creative contributions, nor in how creative other people assess these participants to be.

#### Analysis

Of the 151 participants, 68 were excluded because they did not answer question five, which was the focus of our analysis. Five additional questionnaires were excluded because the raters were unable to understand the participants’ answers. This left a total of 78 participants (56 females and 22 males) whose responses were analyzed. Nearly 58% of the participants were age 50 or older, and half of them worked in art/design. The participants provided a total of 298 influences, and the average number of influences provided by each participant was 3.82 (*SD* = 2.29, *Mdn* = 3.00, range: 1–10).

Two naïve raters independently categorized these responses as either WN, WB, C, or U. The raters were undergraduate psychology students who were blind to the hypothesis behind this study. One was recruited by voluntarily responding to an online recruitment poster listed in UBC Okanagan’s Psychology Course Union website, and the other was recruited through the same poster in a Facebook group for a psychology research methods course. Initially, the raters were provided with detailed instructions and examples for rating the listed influences. These instructions and examples are provided in the Supplementary Materials. The examples provided not only the rating that would be given for an example response, but the reasoning that would lead to the coding decision. The four category definitions given to the raters are provided in Table 2.

**TABLE 2:** Definitions of categories of influence (Studies 1 and 2).

Within-domain narrow (WN):	When the domain of the creative inspiration and the creative output are from the same subcategory, mark the inspiration as WN (e.g., when a painting was inspired by another painting, or a song was inspired by a different song).
Within-domain broad (WB):	When the domain of the creative inspiration is in the same category as the creative output, but not the same subcategory, mark the inspiration as WB [e.g., when a painting is inspired by a photograph (both in the category of visual arts), or when a song is inspired by a musician (the influence is clearly related to the output, but not the same thing)].
Cross-domain (C):	When the inspiration is unrelated to the category of the creative output, mark the inspiration as C (e.g., when a software program is inspired by a song, or when a story is inspired by an emotion).
Unclear (U):	When there is not enough information given to determine whether the influence is within or cross domain, mark the inspiration as U [e.g., when celebration of recovery from injury inspires a dance (it could be within domain if the injury, a physical limitation, is seen as related to the physical movement of dance, or it could be cross domain if the emotion of celebration is seen as something unrelated to the category of performance)].

The raters were then asked to categorize 10 responses, which were reviewed to ensure that they understood the task, before proceeding to code the rest of the questionnaires. The percentage of agreement between the two raters was 86.24%. Cohen’s kappa for the two raters was moderate, κ = 0.58, but improved when partial weights were assigned to the WN/WB categories to increase the tolerance for disagreement when both raters assigned the influence to a within-domain category, κ = 0.64, showing substantial agreement (Landis and Koch, 1977). The raters resolved all disagreements through a phone-call discussion, so that each influence was assigned to only one category. After discussion, raters were more likely to have coded each response as C, WN, or WB, and rarely used the U category.

### Results

As shown in Table 2, the frequency of cross-domain influences (80.54%) was vastly greater than that of within-domain influences, even when both broad and narrow within-domain influences were considered (17.78%). Of the 78 participants, 76 (97.44%) provided at least one cross-domain inspiration in their answer. This indicates that cross-domain sources of inspiration occur frequently for highly creative individuals.

Since participants were encouraged to list all of the influences they could think of that inspired their creative outputs, there was variation in the number of influences that each participant provided. A possible explanation for the low frequency of WN influences (7.72%) could be that there is often only one WN influence that can match a given creative output, but there is a larger number of WB influences, and a potentially infinite number of C influences. Thus, once someone has provided an influence that matches the domain of the creative output, any subsequent influences they provide are more likely to be either cross-domain or within-domain broad. To explore the possibility that our results gave more weight to participants who reported multiple influences, we also analyzed the data according to the number of influences provided by each participant. The breakdown of data by the participants who gave the same number of influences (Table 3) shows that this was not driving the pattern of results. Cross-domain inspiration remained consistently at or above 68% for every number of influence provided. Therefore, our results show a consistently greater occurrence of cross-domain influences than within domain influences, and this result is not dependent on the number of influences provided by a participant. The result of a chi-square test conducted for the result of those who gave only one influence showed that the difference in frequency of each category was significant, χ^2^(3) = 45, *p* < 0.001. Due to the non-independence of the data, no significance testing was conducted for the differences in frequency for those who contributed two or more influences. Instead, we present the frequency of the occurrence of each category for our analysis.

**TABLE 3:** Frequency and percentage of categories of influence by number of influences provided by participant.

		**WN**		**WB**		**C**		**U**	
**No. inf.**	***n***	**f**	**%**	**f**	**%**	**f**	**%**	**f**	**%**
1	15	0	0.0%	0	0.0%	15	100.00%	0	0.0%
2	8	4	25.00%	1	6.25%	11	68.75%	0	0.0%
3	17	7	13.72%	5	9.80%	36	70.59%	3	5.88%
4	14	4	7.14%	2	3.57%	49	87.50%	1	1.79%
5–10	24	8	5.00%	22	13.75%	129	80.62%	1	0.62%
Total (298)	78	23	**7.72%**	30	**10.07%**	240	**80.54%**	5	**1.68%**
95% CI		[4.69, 10.75]		[6.65, 13.48]		[76.04, 85.03]		[0.22, 3.14]	

Table shows the number of participants who provided a given number of influences. 95% confidence interval for total percentages are in square brackets (Study 1). Table shows the occurrence of categories of influence for groups who listed the same number of influences, up to four influences. E.g., in the third row, 17 individuals provided 3 influences, 17^*^3 = 51 influences for that row. No. inf., number of influences; f, frequency; CI, confidence interval; WN, within-domain narrow; WB, within-domain broad; C, cross-domain; U, uncertain. Total number of influences for each category as a percentage of the total number of influences for all categories is indicated in bold.

### Discussion

These results demonstrate that even if individuals primarily express their creativity in a single domain, they are often employing cross-domain thinking when they create. This study enriches our understanding of how the creative process works and adds to the growing body of evidence that creativity is much more than a matter of acquiring domain-specific expertise.

## Study 2

The results of Study 1 prompted speculation as to whether or not the impact of cross-domain influences was limited to individuals with expertise in a particular creative domain. The goal of Study 2 was to assess whether the ability to develop creative works that employ cross-domain influences only comes online after the tools and techniques of a particular domain have been mastered, or whether it generalizes to individuals without expertise in a creative domain. Thus, the study investigated the hypothesis that regardless of one’s expertise in a creative domain, creative outputs will be inspired by cross-domain influences.

### Methods

#### Participants

The sample included 463 undergraduate students (114 males, 347 females, and 2 who selected ‘no or different gender’) from the University of British Columbia. They were recruited through SONA, an online participant pool approved by the UBC Research Ethics Board. Participants were granted one course credit (equal to 1% of their grade) for their participation in this study.

#### Procedure

The SONA website provided a link to an online questionnaire hosted by FluidSurvey. The procedure and questions followed the same format as Study 1.

#### Analysis

Of the 463 participants, 111 were excluded because they left one or more of the written-response questions blank. (They were able to receive course credit whether or not they completed the questionnaire.) An additional 90 questionnaires were excluded because the participants did not provide classifiable answers to question five. Some of the questionnaires were unclassifiable because participants either provided a description of their creative process, or answered with what motivated a creative output, rather than providing an inspiration (e.g., an answer was excluded if the only reason for engaging in the creative activity was that someone else, such as a parent, obliged them to participate). The second category of unclassifiable questionnaires were those in which no creative outputs were provided in conjunction with a creative influence. The last category of unclassifiable questionnaires were those for which the raters found them incomprehensible and impossible to evaluate. This reduced the sample to 262 complete questionnaires for analysis (196 females, 65 males, and one who selected ‘no or different gender’), and these questionnaires provided a total of 758 influences. Each participant listed an average of 2.89 creative influences (*SD* = 1.92, *Mdn* = 3, range: 1–15). Nearly all participants (95.80%) were under the age of 30.

Two naïve raters independently categorized these responses as either WN, WB, C, or U. Both raters were undergraduate psychology students that were recruited using the same strategy employed in Study 1. (One rater assisted with the data from both studies.) The percentage of agreement between the two raters was 78.10%. Cohen’s kappa for two raters was moderate, κ = 0.50, but improved when partial weights were assigned to the WN/WB categories as in Study 1, κ = 0.56 (Landis and Koch, 1977). The raters resolved all disagreements through discussion, so that each influence was assigned to only one category.

A participant’s response to question 1 (their creative domain) was referenced alongside the types of output they recorded in question 5 in order to assign a general creative domain to each participant. We used nine categories in total. Participants who reported two or more outputs from different creative domains were categorized as ‘Multiple Domains.’ ‘Sport’ included dance and gymnastics, as well as team sports. ‘Art’ included sculpting, painting, and drawing. ‘Design’ included different types of design, such as fashion design, and interior design. ‘Other’ included those who did not fit into another category, such as ‘Law’ or ‘Administration.’

### Results

The total number of influences in each category of influence, as well as the distribution among participants who gave the same number of influences, are provided in Table 4. The participants’ creative outputs came from a variety of domains, including drawing, architecture, photography, scientific experiments, song writing, furniture design, biochemistry, and athletic performance. They also gave a wide variety of inspirational sources, ranging from people in their lives such as family, friends, and strangers, to African safaris and The Book of Kells.

**TABLE 4:** Frequency and percentage of categories of influence by number of influences provided by participant.

		**WN**		**WB**		**C**		**U**	
**No. inf.**	***n***	**f**	**%**	**f**	**%**	**f**	**%**	**f**	**%**
1	68	17	25.00%	5	7.35%	44	64.71%	2	2.94%
2	59	20	16.95%	14	11.86%	84	71.19%	0	0%
3	57	21	12.28%	23	13.45%	126	73.68%	1	0.58%
4	40	20	12.50%	15	9.38%	123	76.88%	2	1.25%
5	18	11	12.22%	9	10.00%	70	77.78%	0	0%
6–15	20	18	11.92%	15	9.93%	117	77.48%	1	6.62%
Total (758)	262	107	**14**.**12%**	81	**10**.**69%**	564	**74**.**41%**	6	**0**.**79%**
95% CI		[11.64, 16.59]		[8.49, 12.88]		[71.30, 77.51]		[0.16, 1.42]	

Table shows the number of participants who provided a given number of influences. 95% confidence interval for total percentages are in square brackets (Study 2). Table shows the occurrence of categories of influence for groups who listed the same number of influences, up to five influences. E.g., in the third row, 57 individuals provided 3 influences, 57^*^3 = 171 influences for that row. No. inf., number of influences; f, frequency; CI, confidence interval; WN, within-domain narrow; WB, within-domain broad; C, cross-domain; U, uncertain. Total number of influences for each category as a percentage of the total number of influences for all categories is indicated in bold.

The results were highly consistent with those of Study 1. Of the 758 influences provided, 107 (14.12%) were WN, 81 (10.69%) were WB, and 564 (74.41%) were C. Thus, cross-domain influences constituted three times the number of within-domain influences, even when broad as well as narrow within-domain influences were included (24.80%). Of the 262 participants, 222 of them (84.73%) provided at least one cross-domain influence. Similar to Study 1, regardless of the number of influences provided, at least 64% of them were cross-domain. A chi-square test for the result of those who gave one influence showed that the difference in frequency of each category was significant, χ^2^(3) = 64.59, *p* < 0.001.

Table 5 presents the occurrence of the different categories of influence for individuals in different creative domains, arranged from the highest frequency of cross-domain influences to the lowest. Although comparisons should be made cautiously due to large differences in the sizes of the subsamples, the results suggest that cross-domain influences are slightly less common in Sport and Drama (58.54% and 58.33%, respectively), and most frequent for Writers and Artists (88.89% and 77.93%, respectively). However, it is important to note that for every category of creativity assessed in our sample, cross-domain influences were reported more frequently than any within-domain sources of inspiration.

**TABLE 5:** Frequency (and percentage by row) of categories of influence by creative domain (Study 2).

	**WN**	**WB**	**C**	**U**
Writing	4 (5.56%)	4 (5.56%)	64 (88.89%)	0 (0%)
Art	34 (11.72%)	29 (10.00%)	226 (77.93%)	1 (0.03%)
Multiple domains	13 (11.61%)	16 (14.28%)	83 (74.11%)	0 (0%)
Science	12 (18.75%)	6 (9.38%)	46 (71.88%)	0 (0%)
Music	25 (18.94%)	13 (9.85%)	91 (68.94%)	3 (2.27%)
Design	4 (18.18%)	3 (13.64%)	15 (68.18%)	0 (0%)
Other	3 (23.07%)	2 (15.38%)	8 (61.54%)	0 (0%)
Sport	10 (24.39%)	7 (17.07%)	24 (58.54%)	0 (0%)
Drama	2 (16.67%)	1 (8.33%)	7 (58.33%)	2 (16.67%)

*WN, within-domain narrow; WB, within-domain broad; C, cross-domain; U, uncertain.*

One possible reason for the high number of cross-domain influences for artists and writers is that in these domains creative expression is highly valued, while works that are closely related to previous books/paintings may be derided for their lack of creativity. Moreover, individuals may be specifically drawn to these domains because this kind of expression is accepted and even encouraged. In other domains, it may be more important to draw upon the ideas and methods of previous creators, resulting in more within-domain influences. Cross-domain influences for ‘Sport’ in our sample may be due to dancing being included in this category, and common influences for dance included music, lyrics, and emotions (all cross-domain). Future studies with more complete and representative samples of different creative domains may be able to examine the distinctions within these domains more closely (e.g., to distinguish fiction from poetry or research papers, or to separate dance from soccer).

### Discussion

**FIGURE 1:**
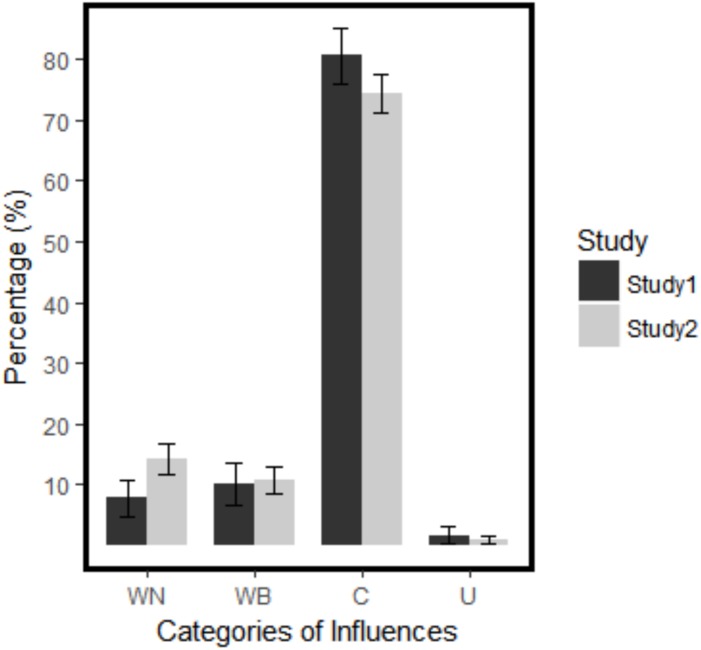
Percentages of the categories of influence in Study 1 and Study 2. Error bars represent 95% confidence interval. WN, within-domain narrow; WB, within-domain broad; C, cross-domain; U, uncertain.

The results of this study concur with those of Study 1 in that the majority of creative outputs were inspired by cross-domain influences. In addition, Study 2 shows that this is not only the case for individuals with proven success or expertise in a creative domain, but holds true for non-experts as well. Except for WN, the confidence intervals from the two studies overlap, indicating that there is no significant difference between the results of Study 1 and Study 2 (see Figure 1).

## General Discussion

### Implications for a Theoretical Framework for Creativity

This result has implications for our understanding of the creative process, because it demonstrates that it is substantially less domain-specific than it is widely presumed to be. Even if individuals primarily express their creativity in a single domain, they are often employing cross-domain thinking when they create. Although domain-specific knowledge may ensure that tools of the trade are appropriately applied, and one’s creative works may consistently be in one particular domain, the sources that initially triggered these creative processes may be diverse in nature. To the extent that this is the case, creativity may involve synthesizing information from different arenas of one’s life. This is consistent with high levels of functional connectivity amongst the default, salience, and executive neural circuits in creative individuals during creative thinking (Beaty et al., 2018). Although the phenomenon of cross-domain influence in creativity—and by implication, the abstraction and re-expression of abstract forms—may seem obvious to artists, it plays little role in the bulk of psychology, computational creativity, and AI research, in which creativity is commonly treated as heuristically guided search or selection amongst discrete, well-defined states, guided by domain-specific expertise (e.g., Weisberg, 1995; Simonton, 2010). The finding that cross-domain influences are widespread is consistent with HT, according to which it is not just one’s conception of the creative task (or ‘problem domain’) but one’s worldview as an integrated whole that transforms—becoming less fragmented and/or more robust—through immersion in a creative task. Honing entails iteratively viewing the creative task from a new context, which may restructure the internal conception of it, and this restructuring may be amenable to external expression. This external change may in turn suggest a new context, and so forth recursively, until the task is complete. The view that creative honing can bring about sweeping changes to an individual’s second (psychological) level of complex, adaptive structure is consistent with findings that creativity is potentially therapeutic (Barron, 1963; Forgeard, 2013), and that through immersion in a creative task, a more stable image of the world, and one’s own relation to it, can emerge (Pelaprat and Cole, 2011). Thus, it is through the interaction and cross-fertilization of knowledge and ideas that a more integrated understanding of the world is achieved (Gabora, 1998; Gabora and Steel, 2017), and psychological entropy (Hirsh et al., 2012; Gabora, 2017) kept to an acceptable minimum.

### Implications for Cultural Evolution

At first glance it might seem that the basic units of cultural evolution (i.e., the cultural equivalent of the organism in biological evolution) are such things as catchy songs, rituals, or tools. However, the above evidence for the cross-fertilization of different domains suggests that the only way to delineate the cultural lineage of a given idea is to look to the creator’s entire loosely integrated web of knowledge and understandings (i.e., the creative process transforms not just the problem domain but the worldview as a whole). In this way, the inspirational sources—or ‘conceptual parents’—of a sad ballad could include everything from other musicians, to the patter of rain, to the death of a loved one. Thus, creative products don’t just serve practical purposes or provide aesthetic pleasure; they provide tangible external markers of the evolutionary states of the worldviews that generated them. This is consistent with the theory that what evolves through culture is, not creative outputs like songs or tools, but worldviews, with creative outputs as the externally visible ‘excrement’ of this transformation process (Gabora, 2004, 2008, 2013). In short, research into cross-domain influences has implications for not just how the creative process works but for how culture evolves.

As discussed elsewhere, a worldview not only self-organizes in response to perturbations but it is imperfectly reconstituted and passed down through culture (Gabora, 2013). This is because it is not just self-organizing but self-regenerating: people share experiences, ideas, and attitudes with each other, thereby influencing the process by which other worldviews form and transform. Children expose elements of what was originally an adult’s worldview to different experiences, different bodily constraints, and thereby forge unique internal models of the relationship between self and world. In short, worldviews transform by interleaving (1) internal interactions amongst their parts, and (2) external interactions with others, and it is through these social interactions that novelty accumulates, and culture evolves.

### Limitations and Future Directions

We attribute the large fraction of respondents who did not complete the questionnaire to the anonymous nature of the study. In future studies, it would be helpful to consider ways of further incentivizing participants to complete the questionnaire. Although the open-ended nature of the questionnaire was necessary to enable participants to provide anything as an inspirational source, it may have been less inviting for those who prefer a structured format. Changing the format from an online questionnaire to an in-person interview may elicit a higher response rate. In addition, the instructions should actively discourage participants from providing motives (e.g., a desire to be creative) instead of inspirational sources, and examples of each should be given so that the distinction is clear. Also, reformatting the questionnaire so that it is always clear which creative influence is associated with which creative output would help clarify the interpretation of the responses.

A limitation of the self-report format is that participants might not accurately remember, or report the inspirations for their creative works. Related to this is that the open-ended nature of participants’ responses meant that they varied in detail and in the extent to which they actually responded to the particular question that was asked. However, we believe the methods chosen matched the goals for this study. There is no easy or standardized way of examining the creative influences of others. Our goal was to obtain the most information from each participant possible by allowing them to respond in their own words, with as much detail as possible, rather than to limit their responses by a more formalized structure such as a survey. Our study showed that when people self-report their creative inspirations, cross-domain influences are frequently reported. Besides the above-mentioned ideas, other possible future methods could include the identification of a creative output, and then to approach the creator for an explanation of their influences for that particular output. This study also made no effort to measure or control for the creativity of the outputs, but this variable could be explored in future studies, to assess its relation to the domains of influence. Another suggestion for future work would be to add a second, within-domain example to the instructions, such as “My Sonata was influenced by my recent obsession with Beethoven’s Pathetique.”

We note as another limitation that the gender balance was skewed toward females, and Study 1 participants tended to be older than Study 2 participants. The age difference arose largely from the difficulty of finding accomplished creative professionals under age 30. In future studies it would be advisable to endeavor to obtain a more equal gender balance and age distribution to ensure that these factors do not affect the results.

An important direction for future research is to investigate the timing and underlying mechanisms of cross-domain influence. In the studies reported here, participants were prompted to focus on the inspiration for their creative outputs, but we note that cross-domain influences are not necessarily exclusive to the inspiration phase of the creative process. To better understand the frequency and timing of cross-domain thinking in the creative process, further research needs to be done on other phases of the creative process, and the process as a whole.

In regards to the mechanisms of cross-domain influence, Boccia et al. (2015) provide evidence that creativity and divergent thinking in visual, musical, and verbal domains are underpinned by specific brain areas, but also share a common brain system. This suggests that our cross-domain results may be due to a ‘general’ cognitive process or brain structure that people use while creating, regardless of domain. However, since Boccia et al. (2015) did not differentiate between the initial creative inspiration and other phases of the creative process (e.g., execution), it is difficult to fully assess the relevance of Boccia et al.’s (2015) analysis to the results reported here.

Another direction is to investigate the hypothesis that individuals in some creative domains make less use of cross-domain influences than others (e.g., scientists use less cross-domain influences than artists), and explore the effect of different types of influence on the level of creative output within that domain. For example, within-domain influences may be important for a field such as science, where domain-specific knowledge is essential (Bermejo et al., 2016), but cross-domain influences may be associated with greater creativity within this domain. Further research could also investigate developmental differences in the ability to employ cross-domain influences. Future research could investigate the factors that predispose individuals to employ cross-domain influences, such as their implicit conception of how the creative process works. Another direction for future research is to investigate the extent to which the application of cross-domain influences is associated with personality traits correlated with creativity, such as norm-doubting, high aspiration levels, tolerance of ambiguity, and openness to experience (Eysenck, 1993; Helson, 1996; Martindale and Daily, 1996; Batey and Furnham, 2006). If so, this would suggest that propensity toward cross-domain influence plays a mediating role between personality and creativity.

### Practical Implications

The finding that the majority of creative outputs were inspired by cross-domain influences has implications for the development of practices that promote creativity in education, the workplace, and personal life. There is an international trend toward streamlining classwork and content to the basics, and reducing or eliminating classes in the arts. Educational systems are increasingly geared toward providing much in the way of information but little in the way of examples of, or opportunities to cultivate, creative thinking skills. The results of this study suggest that creativity may be cultivated by interdisciplinary courses, as well as activities that foster connections between different domains, such as painting murals of ecological food chains or writing poetry about science, as well as exposure to such cross-disciplinary cultural outputs.

## Ethics Statement

The method and recruitment email were both approved by the UBC Research Ethics Board at the University of British Columbia (Okanagan Campus). Written consent was obtained from all participants.

## Author Contributions

LG designed the research. NC conducted Study 1. SW conducted Study 2. VS analyzed the studies. VS and LG wrote the manuscript. All authors contributed to the manuscript revision and approved the submitted version.

## Conflict of Interest Statement

The authors declare that the research was conducted in the absence of any commercial or financial relationships that could be construed as a potential conflict of interest.

## Footnotes

1 Portions of these studies were published in conference proceedings. The findings presented in Study 1 include more than twice as many responses as those presented in Gabora and Carbert (2015). The findings presented in both studies differ from that of both conference proceedings papers (Gabora and Carbert, 2015; Scotney et al., 2018) because the data were subsequently recoded using naïve judges who were blind to the hypothesis of this study (i.e., that cross-domain influences would be widespread).
