# Supplementary material for: The Ubiquity of Cross-Domain Thinking in the Early Phase of the Creative Process
Source: Front Psychol. 2019 Jun 19;10:1426. doi: 10.3389/fpsyg.2019.01426 (PMC6594204; doi:10.3389/fpsyg.2019.01426)
Supplement: Supplementary file 1 [file Data_Sheet_1.PDF]

Instructions Provided to Raters

Creative Influences Study

In this study, we are examining the things that people describe as *influencing* their creative works. By influence, we mean something that they saw or experienced that sparked a creative idea or product. An influence can be something very similar to the creative output, within the same creative domain (e.g., seeing a drawing that inspired a similar drawing), or it can be vastly different (e.g., a camping trip with friends that inspired a screen play). Your task is to identify the domain that an influence comes from.

Please read question 9 of each survey carefully (the one that asks: “Describe all elements that have inspired your work…”), and identify all items mentioned that influenced creative output. You will have access to a list, already created (in the excel sheet provided), of the influences from the surveys, but you may disagree with what we have listed, and add another item to the list if you find one you think was missed. You may not review the surveys in the same order as the list, so please search for the correct entry using key words.

The influences you identified should then be categorized into one of four categories. In order to determine what category it belongs in you may need to find other information in the survey referring to the creative output(s) (question 7). Please indicate the category that best fits the influence in the box next to your listed influence using the letter code provided in brackets.

The four categories:

1. **Within-Domain Narrow (WN):** when the domain of the creative inspiration and the creative output are from the same subcategory, mark the inspiration as WN (e.g., when a painting was inspired by another painting, or a song was inspired by a different song).

2. **Within-Domain Broad (WB):** when the domain of the creative inspiration is in the same category as the creative output, but not the same subcategory, mark the inspiration as WB (e.g., when a painting is inspired by a photograph (both in the category of visual arts), or when a song was inspired by a musician (the influence is clearly related to the output, but not the same thing)).
3. **Cross-Domain (C):** when the inspiration is unrelated to the category of the creative output, mark the inspiration as C (e.g., when a software program is inspired by a song, or when a story is inspired by an emotion).
4. **Unclear (U):** when there is not enough information given to determine whether the influence is within or cross domain, mark the inspiration as U (e.g., when celebration of recovery from injury inspires a dance (it could be within domain if the injury, a physical limitation, is seen as related to the physical movement of dance, or it could be cross domain if the emotion of celebration is seen as something unrelated to the category of performance)).

When to EXCLUDE an item: Any item that *motivated* a creative activity, rather than inspiring it, should be excluded. For example, if the only reason for engaging in the creative activity was that someone else obliged them to participate (e.g., My dad made me take piano lessons), or that they had a general interest in the topic (e.g., My soccer skills are inspired by a love of the sport), then the answer should be excluded. If no creative *output* is specified, the influence should not be included because it is not possible to determine whether the inspirational stimuli belonged to a similar (Within) or dissimilar (Cross) domain. Finally, if an answer does not include an inspiration, but instead is a description of how a creative product was performed or created, the answer should not be included.
